# Synthesis of Nanoscale CaO-Al_2_O_3_-SiO_2_-H_2_O and Na_2_O-Al_2_O_3_-SiO_2_-H_2_O Using the Hydrothermal Method and Their Characterization

**DOI:** 10.3390/ma10070695

**Published:** 2017-06-26

**Authors:** Jingbin Yang, Dongxu Li, Yuan Fang

**Affiliations:** 1 Jiangsu National Synergetic Innovation Center for Advanced Materials (SICAM), Nanjing Tech University, Nanjing 210009, China; yangjingbin@njtech.edu.cn (J.Y.); dongxuli@njtech.edu.cn (D.L.); 2 Guangdong Provincial Key Laboratory of Durability for Marine Civil Engineering, College of Civil Engineering, Shenzhen University, Shenzhen 518060, China

**Keywords:** C-A-S-H, N-A-S-H, hydrothermal method, alkali-activated materials, micro-structure characterization

## Abstract

C-A-S-H (CaO-Al_2_O_3_-SiO_2_-H_2_O) and N-A-S-H (Na_2_O-Al_2_O_3_-SiO_2_-H_2_O) have a wide range of chemical compositions and structures and are difficult to separate from alkali-activated materials. Therefore, it is difficult to analyze their microscopic properties directly. This paper reports research on the synthesis of C-A-S-H and N-A-S-H particles with an average particle size smaller than 300 nm by applying the hydrothermal method. The composition and microstructure of the products with different CaO(Na_2_O)/SiO_2_ ratios and curing conditions were characterized using XRD, the RIR method, FTIR, SEM, TEM, and laser particle size analysis. The results showed that the C-A-S-H system products with a low CaO/SiO_2_ ratio were mainly amorphous C-A-S-H gels. With an increase in the CaO/SiO_2_ ratio, an excess of Ca(OH)_2_ was observed at room temperature, while in a high-temperature reaction system, katoite, C_4_AcH_11_, and other crystallized products were observed. The katoite content was related to the curing temperature and the content of Ca(OH)_2_ and it tended to form at a high-temperature and high-calcium environment, and an increase in the temperature renders the C-A-S-H gels more compact. The main products of the N-A-S-H system at room temperature were amorphous N-A-S-H gels and a small amount of sodalite. An increase in the curing temperature promoted the formation of the crystalline products faujasite and zeolite-P. The crystallization products consisted of only zeolite-P in the high-temperature N-A-S-H system and its content were stable above 70%. An increase in the Na_2_O/SiO_2_ ratio resulted in more non-bridging oxygen and the TO_4_ was more isolated in the N-A-S-H structure. The composition and microstructure of the C-A-S-H and N-A-S-H system products synthesized by the hydrothermal method were closely related to the ratio of the raw materials and the curing conditions. The results of this study increase our understanding of the hydration products of alkali-activated materials.

## 1. Introduction

Alkali-activated materials are an environmentally-friendly building material that reduces CO_2_ emissions by 50% to 80% compared to Portland cement [1] and the materials have high strength, good corrosion and fire resistance, and other excellent performance characteristics, with good prospects for further development. In a high-calcium system (such as blast furnace slag), the hydration products of alkali-activated materials are CaO(-Al_2_O_3_)-SiO_2_-H_2_O-type gels (C(-A)-S-H) [2,3,4]. C-A-S-H gels can be regarded as a new phase of aluminum in the C-S-H structure and it is generally believed that the C-A-S-H gels and the C-S-H gels have a similar tobermorite structure [5,6]. In a low-calcium system (such as low-calcium fly ash and metakaolin), the hydration products of alkali-activated materials are generally Na_2_O-Al_2_O_3_-SiO_2_-H_2_O (N-A-S-H)-type gels [5]. It has been confirmed that the N-A-S-H gels consist of a three-dimensional network structure formed by an aluminum oxygen tetrahedron and a silicon oxygen tetrahedron through the sharing of O atoms, and that the chemical composition and structure of N-A-S-H gels are similar to zeolites; therefore, the structure is also called a zeolite-like structure [7].

Because the main raw materials of alkali-activated materials are mostly industrial by-products with variable chemical compositions [5], the chemical composition of the raw materials has a significant effect on the performance of the alkali-activated materials. There are many research studies on the structure and reaction mechanisms of alkali-activated materials but the findings often reveal marked differences [8]. In view of this, several studies have used SiO_2_ and Al_2_O_3_ and other high-purity raw materials with accurate stoichiometric control instead of slag and fly ash and other industrial by-products to investigate the alkali-activated reaction process. Walkley et al. [9,10] synthesized a precursor powder to replace the fly ash and slag with SiO_2_, Al(NO_3_)_3_∙9H_2_O, and Ca(NO_3_)_2_∙4H_2_O via an organic steric entrapment route and utilizing polyvinyl alcohol and polyethylene glycol as polymeric carriers. L’Hôpital, et al. [11,12] synthesized CaO∙Al_2_O_3_ (CA) by calcining Al_2_O_3_ and CaCO_3_ and then mixing CA, CaO, and SiO_2_ in water in accordance with Ca/Si and Al/Si to obtain C-A-S-H gels. Garcı’a-Lodeiro et al. [13,14] and Xue-min et al. [15] used Na_2_SiO_3_ and tetraethylorthosilicate as the silicon source, respectively, and Al(NO_3_)_3_∙9H_2_O as the aluminum source to synthesize a geopolymer via the sol-gel method.

As C-S-H gels are the main sources of strength for Portland cement, C-A-S-H gels and N-A-S-H gels also play an important role in alkali-activated materials; however, because of the complexity and diversity of the hydration products, it is difficult to separate the high-purity C-A-S-H and N-A-S-H products from the alkali-activated materials and it is not easy to characterize the structure and composition of C-A-S-H and N-A-S-H. Researchers have synthesized C-S-H gels with relatively high purity by the sol-gel method [14], the precipitation method [16], and the hydrothermal method [17,18]. However, in the process of synthesizing C-S-H, C-A-S-H, and other hydration products by the sol-gel method and the precipitation method, it is inevitable to introduce NO^3−^ and Cl^−^ ions. The hydrothermal synthesis of C-A-S-H and N-A-S-H can accurately control the composition of the raw materials and does not introduce other impurity ions, making it easy to analyze and characterize the properties of C-A-S-H and N-A-S-H. In this study, the alkali-activated aluminosilicate products C-A-S-H and N-A-S-H were synthesized by the hydrothermal method using nano-SiO_2_ and nano-Al_2_O_3_ as the main raw materials. The products were characterized by X-ray diffraction (XRD), Fourier transform infrared spectroscopy (FTIR), scanning electron microscopy (SEM), transmission electron microscopy (TEM), and laser particle size analysis to analyze the effects of the CaO(Na_2_O)/SiO_2_ molar ratios, the reaction temperature, and the reaction time on the product composition and microstructure. The results can approximately reflect the changes in the composition and structure of the alkali-activated materials with regard to temperature, time, and composition of the raw materials and can promote further research on alkali-activated materials.

## 2. Experimental Procedures

### 2.1. Materials

Nano-SiO_2_ (50 ± 5 nm) (Macklin Biochemical Co., Shanghai, China) and nano-Al_2_O_3_ (γ-phase, 10 nm) (Macklin Biochemical Co., Shanghai, China) were used as the sources of silicon and aluminum and the other materials included Ca(OH)_2_ (Xilong Scientific Co., Shantou, China), NaOH (Xilong Scientific Co., Shantou, China), anhydrous ethanol (Xilong Scientific Co., Shantou, China), and deionized water (Hugke, Shenzhen, China).

### 2.2. Synthesis of C-A-S-H and N-A-S-H

The mixing proportions of nano-SiO_2_, nano-Al_2_O_3_, Ca(OH)_2_, NaOH, and deionized water are shown in Table 1. Nano-SiO_2_ and nano-Al_2_O_3_ were added to deionized water successively, mixing the product each time by ultrasonic dispersion for 3 min, followed by the addition of Ca(OH)_2_ or NaOH. The raw materials were mixed and heated at different temperatures (room temperature, 60 °C, and 95 °C) in a water bath and were stirred for 90 min; this process was conducted in an N_2_ atmosphere to reduce the impact of CO_2_ in the air. Subsequently, the mixture was sealed in a polytetrafluoroethylene container and placed in a water bath at different temperatures (room temperature, 60 °C, and 95 °C). After curing to a certain age (1 day, 3 days, and 7 days), the reaction product was removed, washed twice with deionized water and absolute ethanol, and then was suction-filtrated and vacuum-dried to obtain C-A-S-H and N-A-S-H samples. In this paper, using CASH1.0_R_1d as an example, CASH1.0 indicates that the CaO/SiO_2_ ratio in the C-A-S-H system is 1.0, R indicates that the curing temperature is room temperature, and 1d indicates that the curing time is 1 day.

### 2.3. Characterization

X-ray diffraction (XRD) measurements were performed using a D8 Advance diffractometer (Brucker, Karlsruhe, Germany) with a copper target, λ = 1.5418 Å, 40 kV, 40 mV. The scan range was 5°–80°, 0.02°/step. C-A-S-H and N-A-S-H powder samples were dried at 50 °C for 48 h in a vacuum environment. Scanning electron microscopy (SEM) was conducted using a Quanta TM 250FEG instrument (FEI Company, Hillsboro, OR, USA) with a 20 kV accelerating voltage and a working distance of 10 mm. The C-A-S-H and N-A-S-H powder samples were coated with 20 nm of gold to make them conductive. Fourier transform infrared spectroscopy (FTIR) was conducted using a Spectrum One Version B spectrophotometer (PerkinElmer, Boston, MA, USA). The specimens were prepared by mixing 1 mg of sample with 300 mg of KBr. The range of spectral analysis was 4000–400 cm^−1^ at a resolution of 4 cm^−1^. Transmission electron microscopy (TEM) was performed using a JEM-1230 instrument (JEOL, Tokyo, Japan) with an 80 kV accelerating voltage. The samples for TEM were prepared by dispersing the C-A-S-H and N-A-S-H powders in ultra-high purity ethanol. A drop of the suspension was then allowed to evaporate on a holey carbon film supported by a 300 mesh copper TEM grid. The particle size distribution of the C-A-S-H and N-A-S-H powder samples was analyzed with a DelsaMax CORE nanolaser particle size analyzer (Beckman Coulter, Shanghai, China). The powder samples were ultrasonically dispersed in deionized water for 5 min based on the ratio of 0.3 g/L and the suspension was detected.

## 3. Results and Discussion

### 3.1. XRD and Reference Intensity Ratio (RIR) Analysis

**Figure 1:**
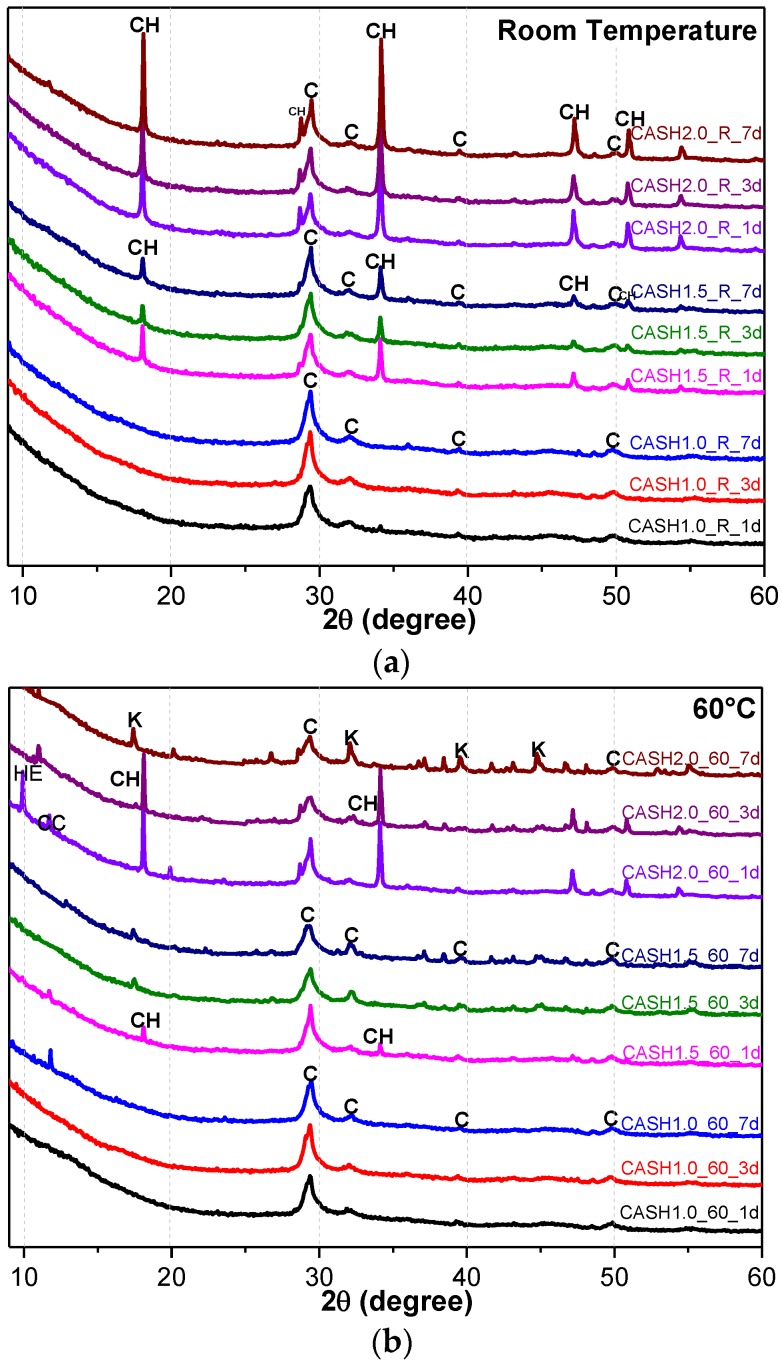

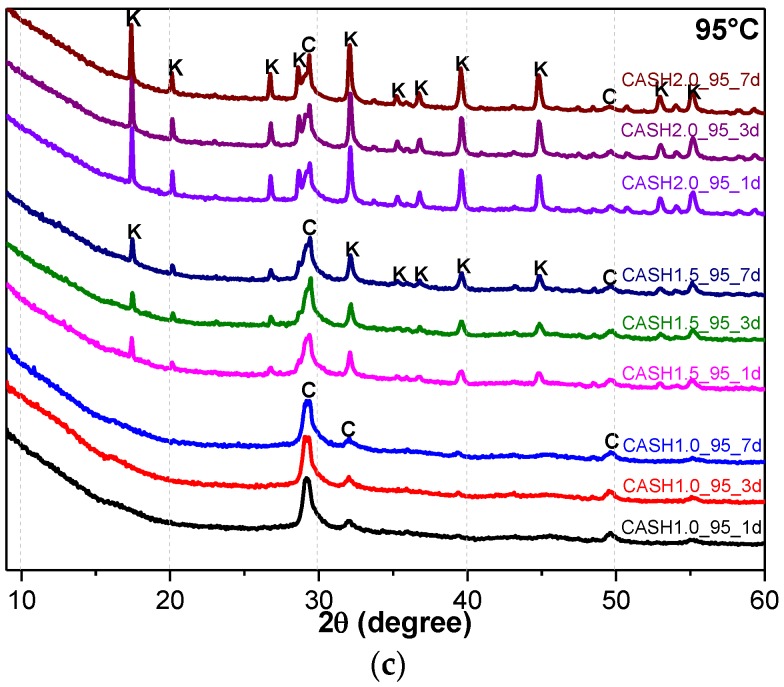
XRD patterns of C-A-S-H system powders; (**a**) reaction at room temperature; (**b**) reaction at 60 °C; (**c**) reaction at 95 °C. (C: C-A-S-H gels; CH: Ca(OH)_2_; K: Katoite; CC: C_4_AcH_11_; HE: Heulandite).

**Figure 2:**
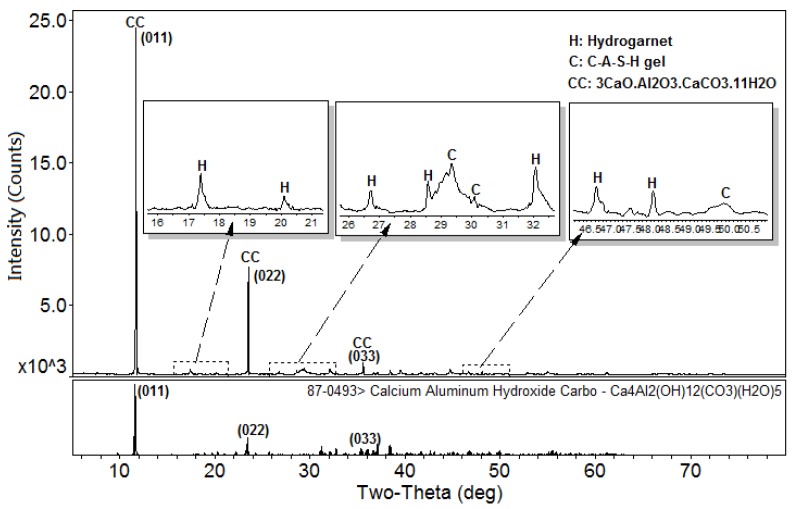
XRD patterns of the C-A-S-H2.0_60_7d powders. (C: C-A-S-H gels; K: Katoite; CC: C_4_AcH_11_).

**Figure 3:**
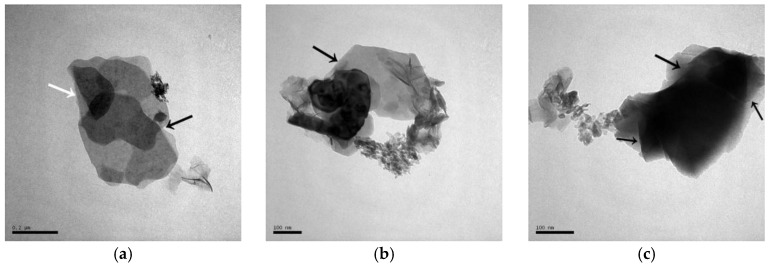
Transmission electron microscopy (TEM) images of C-A-S-H2.0_60_7d. (**a**,**b**,**c**) C-A-S-H2.0_60_7d.

Figure 1 shows the XRD patterns of the C-A-S-H system powders. (CaO/SiO_2_ ratio was 1.0, 1.5, and 2.0; the reaction temperature was room temperature, 60 °C, and 95 °C; the curing period was 1 day, 3 days, and 7 days, respectively, for a total of 27 samples). Figure 1b shows the diffraction peak of calcium monocarboaluminate hydrate (C_4_AcH_11_, PDF#87-0493) when the CaO/SiO_2_ ratio was 1.5 and the reaction temperature was 60 °C. C_4_AcH_11_ has been identified as Na_2_CO_3_-activated slag binders when cured for 1 day and for 540 days [19] but it cannot always be observed due to its poor crystalline structure in an activated slag binder [20]. Although CO_3_^2−^ does not occur in the raw materials and the stirring process was carried out in an N_2_ atmosphere, the CO_3_^2−^ present in the products may be caused by a small amount of CO_2_ dissolved in the water or present in the sealed container during the curing process, and CO_2_ reacted with aluminum oxide in the water bath environment at 60 °C to form C_4_AcH_11_. Figure 2 compares the XRD patterns of sample C-A-S-H_60_7d and the C_4_AcH_11_ normal diffraction card. The XRD patterns of C-A-S-H_60_7d showed strong diffraction peaks at 11.69°, 23.51°, and 35.58° 2θ, corresponding to the (011), (022), and (033) reflections of C_4_AcH_11_. The diffraction peak intensity value at 11.69° 2θ is 26676, however, the diffraction peak of C-A-S-H gels at 29.3° 2θ is only 424, which represents a more than 60-fold difference. In addition, a two-dimensional sheet structure with a size of about 200 nm × 200 nm was detected in this sample by TEM (as shown in Figure 3 in the marked areas with arrows). Thus, these three strong diffraction peaks can be attributed to the orientation growth of the crystals in the crystal planes (011) or the preferred orientation due to the preparation of the XRD samples by the tableting method and the two-dimensional sheet structure observed in the TEM images are associated with the secondary product C_4_AcH_11_. Due to the high intensity of these three diffraction peaks, it is difficult to observe and determine if other phases exist in the samples and the high diffraction peaks at 11.69° and 23.51° 2θ were removed from the XRD patterns of the four samples CASH1.5_60_3d, CASH1.5_60_7d, CASH2.0_60_3d, and CASH2.0_60_7d in Figure 1.

As shown in Figure 1, the diffraction peaks of all C-A-S-H samples with a CaO/SiO_2_ ratio of 1.0 at 29.3°, 32.0°, and 49.8° 2θ were attributed to semi-crystalline aluminum-doped C-A-S-H gels with a tobermorite structure [21,22,23]. At room temperature, as the CaO/SiO_2_ ratio is increased to 1.5 and 2.0, excess Ca(OH)_2_ (PDF#87-0673) can be observed at 18.07°, 34.09°, and 47.13° 2θ in Figure 1a. The C-A-S-H samples obtained by increasing the curing temperature to 60 °C and 95 °C are shown in Figure 1b,c. For the samples with CaO/SiO_2_ ratios of 1.5 and 2.0, other crystallized products such as C_4_AcH_11_, katoite (siliceous hydrogarnet, Ca_3_Al_2_(SiO_4_)_3-*x*_(OH)_4*x*_ with 1.5 ≤ *x* ≤ 3.0, PDF#84-0917), and a small amount of zeolitic products, such as heulandite (Ca_1.23_(Al_2_Si_7_O_18_)(H_2_O)_6_, PDF#85-1386), can be observed. Previous studies [24] have suggested that katoite is present in aged Na_2_SiO_3_-activated slags after 3-year curing as a crystallized C-A-S-H product and that a high content of Si leads to the formation of katoite in activated slag binders. High-temperature hydrothermal conditions can accelerate the formation rate of katoite and cause it to form at an earlier stage compared to alkali-activated slag. Heulandite has been identified as a Ca-containing zeolitic product in silicate-activated slag binders according to XRD analysis [25,26]. The diffraction intensity of heulandite increased during the curing time of 7–56 d, but after 180 d, the diffraction peak almost disappeared. All C-A-S-H system products at room temperature, 60 °C, and 95 °C with a low CaO/SiO_2_ ratio were C-A-S-H gels. This suggests that the C-A-S-H gels was preferred in the low-calcium C-A-S-H reaction system and that the excess Ca(OH)_2_ can form other crystallization C-A-S-H products as the CaO/SiO_2_ ratio increases; in addition, the results indicate that the type and quantity of crystallization products are related to the curing temperature and the curing time.

The katoite phase and the excess Ca(OH)_2_ content in the C-A-S-H product powders were quantitatively analyzed by the reference intensity ratio (RIR) method [27] in order to further explore the reaction degree of the C-A-S-H system and the content of the crystalline phase. The C-A-S-H product powder was homogeneously mixed with α-Al_2_O_3_ with a 1:1 mass ratio and then analyzed by XRD. The strongest diffraction peaks of the quantitative phase and α-Al_2_O_3_ were fitted by using MDI Jade and the intensity was recorded as *I*_i_ and *I*_α-Al2O3_, respectively; the reference intensities *K*_i_ of Ca(OH)_2_ and katoite were 3.50 (PDF#87-0673) and 1.09 (PDF#84-0917), respectively, *W*_α-Al2O3_ = 50%. The content of the quantitative phase in the mixed sample (*W*_i_^1^) can be calculated according to the formula:

(1) $${W_{i}}^{1}=\frac{W_{\alpha -{\mathrm{Al}}_{2}O_{3}}}{K_{i}}\times \frac{I_{i}}{I_{\alpha -{\mathrm{Al}}_{2}O_{3}}}\;,$$

The mass fraction of the quantitative phase in the original samples (not mixed with α-Al_2_O_3_) (*W*_i_) can be calculated according to the formula:

(2) $$W_{i}=\frac{{W_{i}}^{1}}{1-W_{\alpha -{\mathrm{Al}}_{2}O_{3}}}\;,$$

The mass fractions of Ca(OH)_2_ and katoite in each sample are shown in Table 2.

**Figure 4:**
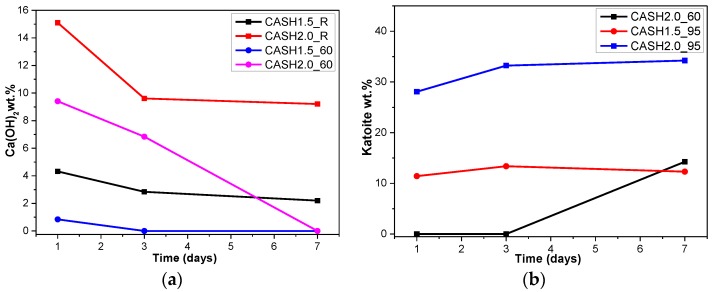
Calcium hydroxide and katoite content on different days. (**a**) Calcium hydroxide; (**b**) Katoite.

Figure 4a reflects the changes in the Ca(OH)_2_ content in different samples. The Ca(OH)_2_ content is related to the curing temperatures, the curing times, and the CaO/SiO_2_ ratio. Ca(OH)_2_ has completely reacted in the C-A-S-H system when the CaO/SiO_2_ ratio is 1.0. However, when the CaO/SiO_2_ ratio was increased to 1.5 and 2.0, the Ca(OH)_2_ in the low-temperature cured C-A-S-H system was excessive. With an increase in the curing time, the excess Ca(OH)_2_ continued to react and the content decreased. The Ca(OH)_2_ in the samples with CaO/SiO_2_ ratios of 1.5 and 2.0 at 60 °C was completely reacted at 3 d and 7 d, however, the excess Ca(OH)_2_ reaction rate of the samples cured at room temperature after 3 d was slow and the content of Ca(OH)_2_ was stable. This indicates that an increase in the curing temperature can significantly accelerate the reaction rate of the C-A-S-H system. Figure 4b shows the change in the katoite content in the C-A-S-H system. Katoite did not appear in the C-A-S-H sample at room temperature and the only sample that contained katoite (14.26 wt.%) was CASH2.0_60_7d at 60 °C. However, when the curing temperature was increased to 95 °C, the katoite content in the samples with a CaO/SiO_2_ ratio of 1.5 was stable in the range of 11–13 wt.% and the katoite contents in CASH1.5_95_3d, CASH1.5_95_7d, CASH2.0_95_3d, and CASH2.0_95_7d were 13.38 wt.%, 12.31 wt.%, 33.23 wt.%, and 34.22 wt.%, respectively. The increase in the curing time did not significantly increase or reduce the katoite content, suggesting that the excess Ca(OH)_2_ had completely reacted so that katoite could no longer be generated. By analyzing the phase composition of all the samples, it was found that the products of siliceous hydrogarnet (Ca_3_Al_2_(SiO_4_)_3−*x*_(OH)_4*x*_ with 1.5 ≤ *x* ≤ 3.0) were more likely to occur in the C-A-S-H reaction system with high calcium and at a high temperature; these results are consistent with those of Rojas et al. [28]. The crystalline products tend to form at high-temperature or high-pressure conditions, and the C-A-S-H gels are amorphous or semi-crystalline, so when there is excessive Ca(OH)_2_ present in the reaction system, crystalline products with a thermodynamic steady state tend to form, rather than forming amorphous gels.

**Figure 5:**
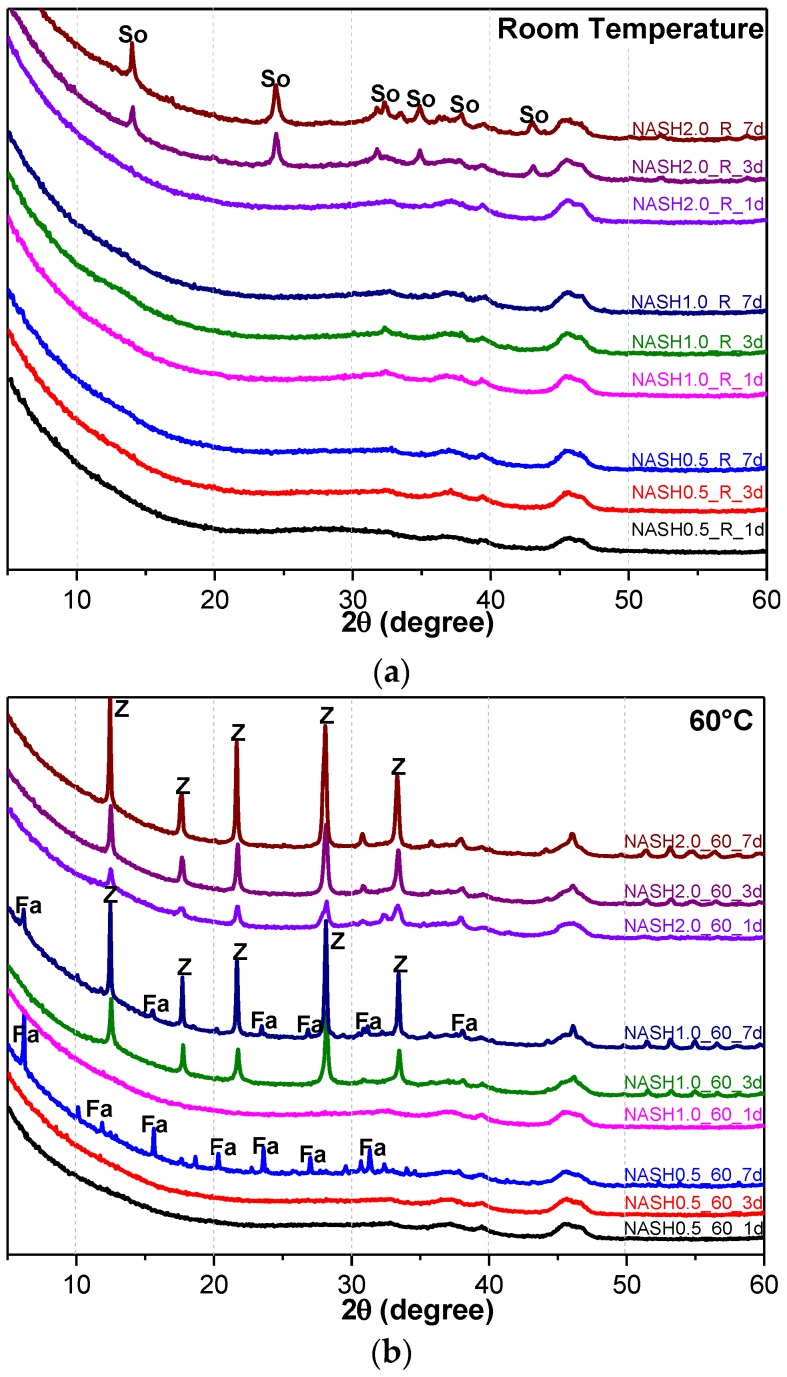

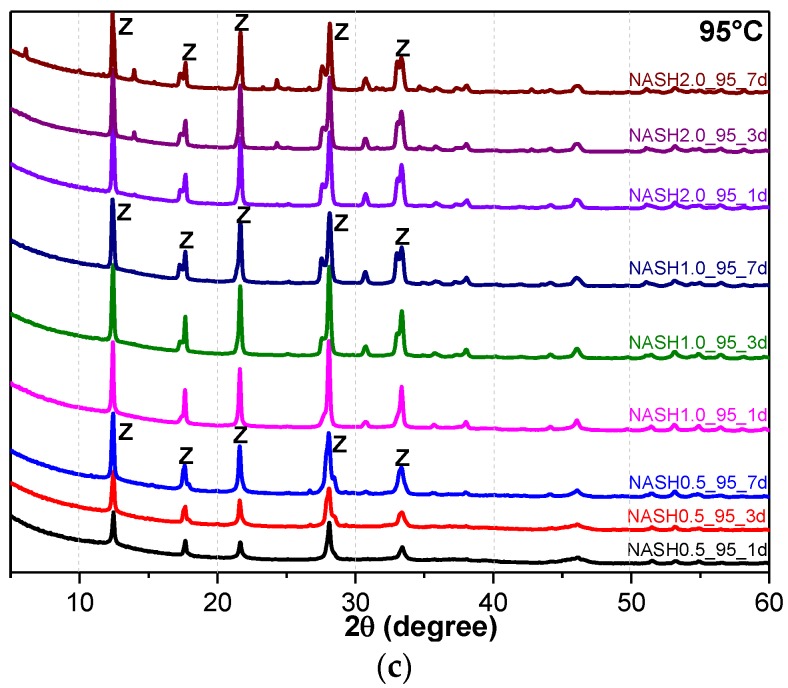
XRD patterns of N-A-S-H system product powders. (**a**) reaction at room temperature; (**b**) reaction at 60 °C; (**c**) reaction at 95 °C. (So: Sodalite; Fa: Faujasite; Z: Zeolite-P).

Figure 5 shows the XRD patterns of the N-A-S-H system product powders. (Na_2_O/SiO_2_ ratio was 0.5, 1.0, and 2.0; reaction temperature was room temperature, 60 °C, and 95 °C; the curing period was 1 d, 3 d, and 7 d, respectively, for 27 samples). The crystallization products sodalite, faujasite, and zeolite-P in the N-A-S-H system were quantitatively analyzed by the RIR method and the reference intensities *K*_i_ were 2.13 (PDF#76-1639), 1.02 (PDF#43-0168), and 1.69 (PDF#44-0052), respectively (Table 3).

**Figure 6:**
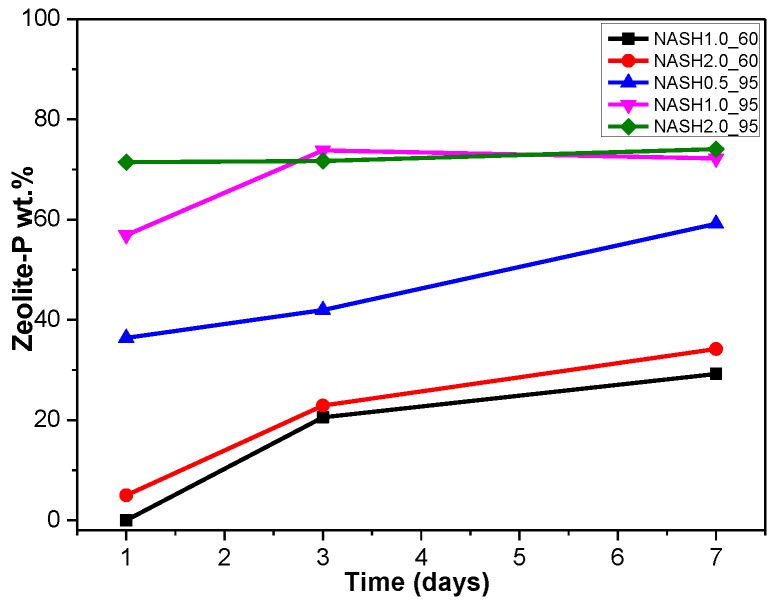
Zeolite-P content on different days.

Figure 5a indicates that the N-A-S-H products at room temperature are mainly amorphous, except that a few diffraction peaks for sodalite (Na_8_Al_6_Si_6_O_24_(OH)_2_(H_2_O)_2_, PDF#76-1639) can be observed in the samples NASH2.0_R_3d and NASH2.0_R_7d; in addition, a broad featureless hump due to amorphous aluminosilicate is observed between 25° and 35° 2θ. Sodalite exists in fly ash-based geopolymers [29,30] and can be considered a zeolitic precursor representing the structure of a geopolymer reaction product. This is similar to the role that 14 Å tobermorite and jennite play in Portland cement research, aimed at understanding the mechanical properties of geopolymers at an atomic level. The compressibility of sodalite is more relevant to the chemical composition of the sodalite framework structure than the topology of zeolite itself [29]. When the curing temperature was increased to 60 °C, the content of faujasite was 11.62 wt.% in NASH0.5_60_7d; 9.81 wt.% in NASH1.0_60_7d, and the zeolite-P content was 29.19 wt.%; the faujasite diffraction peak disappeared in NASH2.0_60_7d and the crystalline phase was all zeolite-P. The content of faujasite did not increase with an increase in the Na_2_O/SiO_2_ ratio but clearly decreased until NASH2.0_60_7d disappeared completely. At a curing temperature of 95 °C, there was no faujasite in any sample, which indicates that the faujasite in a high Na_2_O/SiO_2_ ratio or in a high-temperature environment is converted to zeolite-P. The existence of faujasite has been observed in metakaolin-based geopolymers cured at 65 °C for 3 days and the faujasite formation caused a decrease in the early compressive strength, indicating that the compressive strength was negatively correlated with the faujasite content [31]. The curing conditions that caused faujasite to exist in metakaolin-based geopolymers described in the above-mentioned study were similar to those of the laboratory-synthesized N-A-S-H. Figure 6 shows the change in the zeolite-P content in the N-A-S-H system at 60 °C and 95 °C. The zeolite-P content in the N-A-S-H samples cured at 60 °C was much lower than that of the N-A-S-H samples cured at 95 °C with the same Na_2_O/SiO_2_ ratio, while the zeolite-P content was higher in the high Na_2_O/SiO_2_ ratio system than in the low Na_2_O/SiO_2_ ratio system for curing temperatures of 95 °C. An increase in the curing temperature and the Na_2_O/SiO_2_ ratio can promote the formation of more zeolite-P in the N-A-S-H system. The zeolite-P contents in the samples with Na_2_O/SiO_2_ ratios of 1.0 and 2.0 after 3 days of curing at a high temperature of 95 °C were more than 70% and the zeolite-P content was stable.

### 3.2. Fourier Transform Infrared Spectroscopy

**Figure 7:**
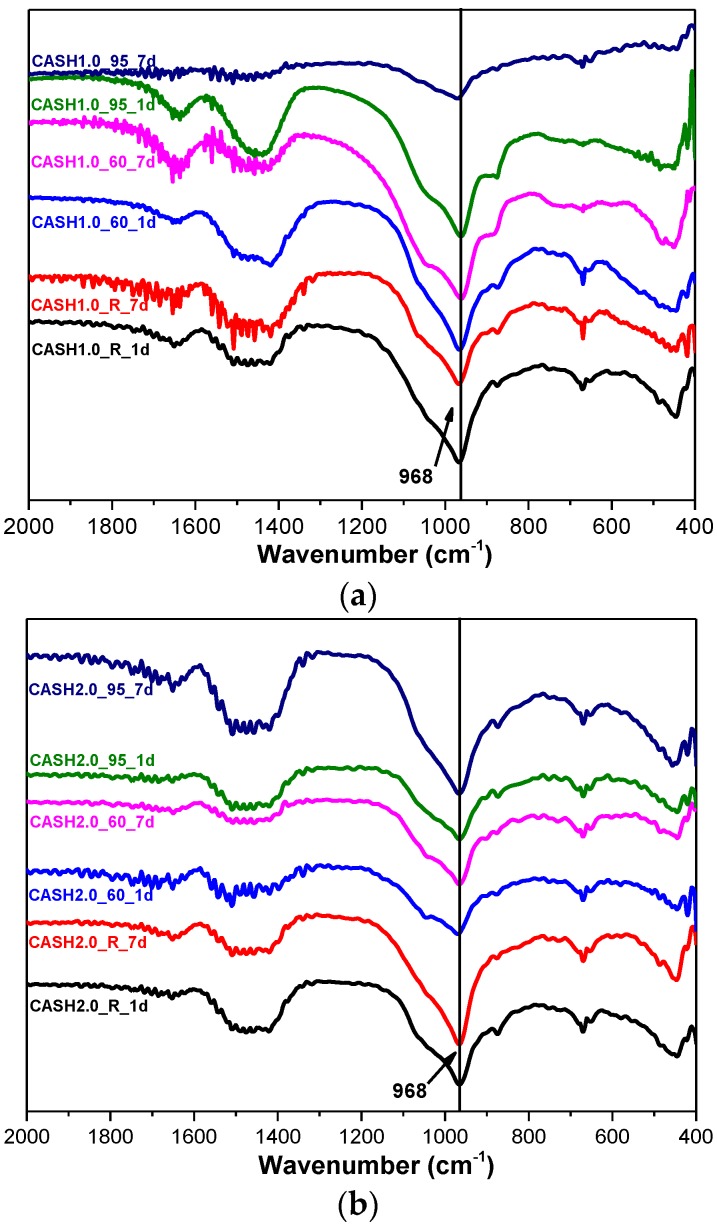
FTIR spectra of C-A-S-H system products. (**a**) CaO/SiO_2_ is 1.0; (**b**) CaO/SiO_2_ is 2.0.

Figure 7 shows the FTIR spectra of the samples for CaO/SiO_2_ ratios of 1.0 and 2.0 at different curing temperatures and curing times. In the range of 2000–400 cm^−1^, the weak bands around 1650 cm^−1^ and 1400 cm^−1^ are attributed to the bending vibrations of OH^−^ in free or chemically bound water and to the stretching vibrations of the C–O bands in CO_3_^2−^ formed by carbonization [14]. The shoulder around 1050 cm^−1^ is due to Si–O–Si of silica in some samples with incomplete reactions [14] and this feature disappeared when silica reacted completely. The band at 968 cm^−1^ is assigned to the asymmetric stretching vibrations of the Si–O–T bonds (T is Si or Al) [9] and the bands at 670 cm^−1^ and 441 cm^−1^ are attributed to the symmetric stretching vibrations of Si–O–T and the bending vibrations of Si–O–Si in SiO_4_, respectively [9,32]. The broad intense band at 968 cm^−1^ and the bands at 670 cm^−1^ and 441 cm^−1^ did not change significantly for all the C-A-S-H samples, indicating that there was no considerable modification in the position of the bridge oxygen bond in TO_4_ with changes in the curing temperature, the curing times, and the CaO/SiO_2_ ratio.

**Figure 8:**
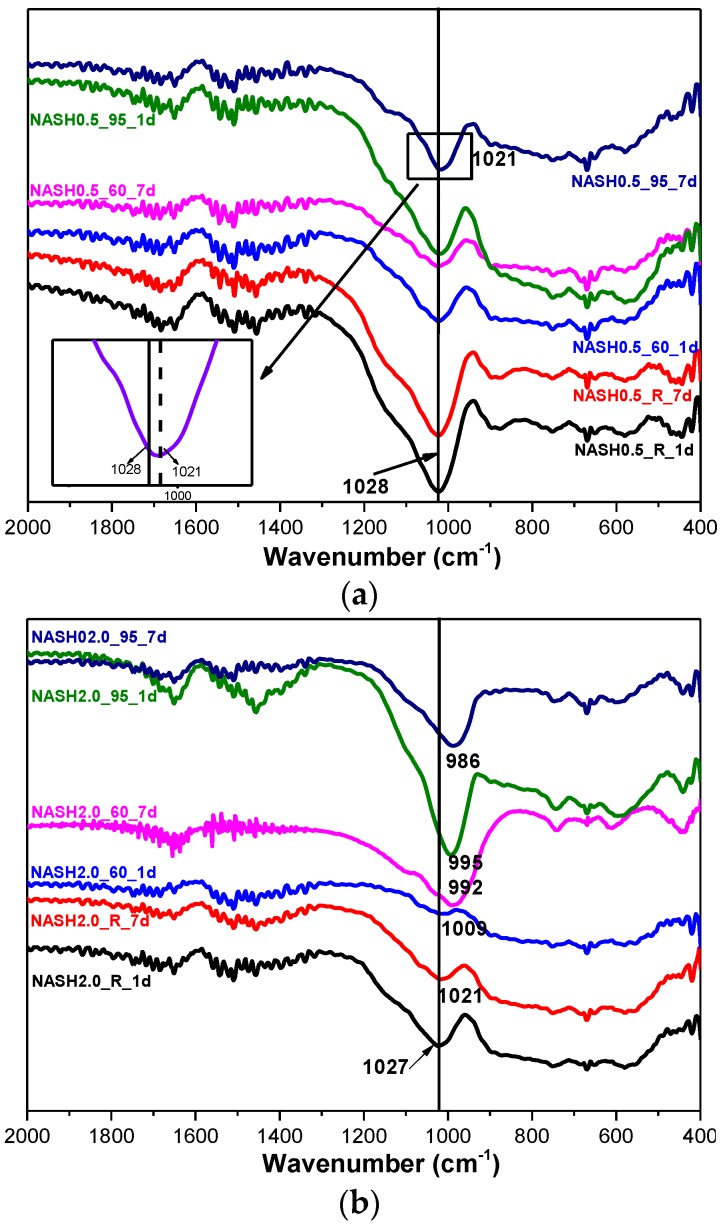
FTIR spectra of N-A-S-H system products. (**a**) Na_2_O/SiO_2_ is 0.5; (**b**) Na_2_O/SiO_2_ is 2.0.

Figure 8 shows the FTIR spectra of the samples with Na_2_O/SiO_2_ ratios of 0.5 and 2.0 at different curing temperatures and curing times. The bands at 1650 cm^−1^ and 1400 cm^−1^ are associated with the bending vibrations of O–H in free or chemically bound water and the stretching vibrations of the C–O bonds for a small amount of Na_2_CO_3_; the Na_2_CO_3_ is caused by carbonization [10,33]. The principal band at 1028 cm^−1^ is attributed to the asymmetric stretching vibrations of Si–O–T (T is Si or Al) in the N-A-S-H system products [10,34,35]. The bands in the range of 800–600 cm^−1^ and 600–400 cm^−1^ are attributed to the stretching vibrations of Al–O and the bending vibrations of Si–O–Si and Si–O–Al, respectively [36,37]. In the low Na_2_O/SiO_2_ ratio products, the band due to the asymmetric stretching vibrations of the Si–O–T bonds is located at 1028 cm^−1^ and it only slightly shifts to 1021 cm^−1^ when the curing temperature is increased to 95 °C and the curing time is extended to 7 days. In the high Na_2_O/SiO_2_ ratio system (Figure 8b), the band shifts to the low wavenumber significantly, and with an increase in the curing temperature and the curing time, this band in sample NASH2.0_95_7d shifts to 986 cm^−1^. A comparison of the positions of the bands attributed to the asymmetric stretching vibrations of T–O–Si for the high Na_2_O/SiO_2_ ratio and the low Na_2_O/SiO_2_ ratio indicates that with an increase in the Na_2_O/SiO_2_ ratio, the amount of Na^+^ in the system is higher than required to achieve an equilibrium with the AlO_4_^−^ tetrahedron. In order to maintain an electric neutrality of the structure, the number of non-bridging bonds with a negative charge in the structure increased and the non-bridging oxygen bonds are not shared by two or more polyhedra, resulting in a greater isolation of the N-A-S-H structure and a lowering of the molecular vibration force constant of the Si–O–T bonds, and the result of this in FTIR is that the band due to the Si–O–T asymmetric stretching shifts toward the lower wavenumbers [10,35]. With an increase in the curing time and the temperature, the band appears at a lower wavenumber, which indicates that more excess Na^+^ in the reaction system enters the aluminosilicate structure and the reaction degree has increased.

### 3.3. Scanning Electron Microscopy

**Figure 9:**
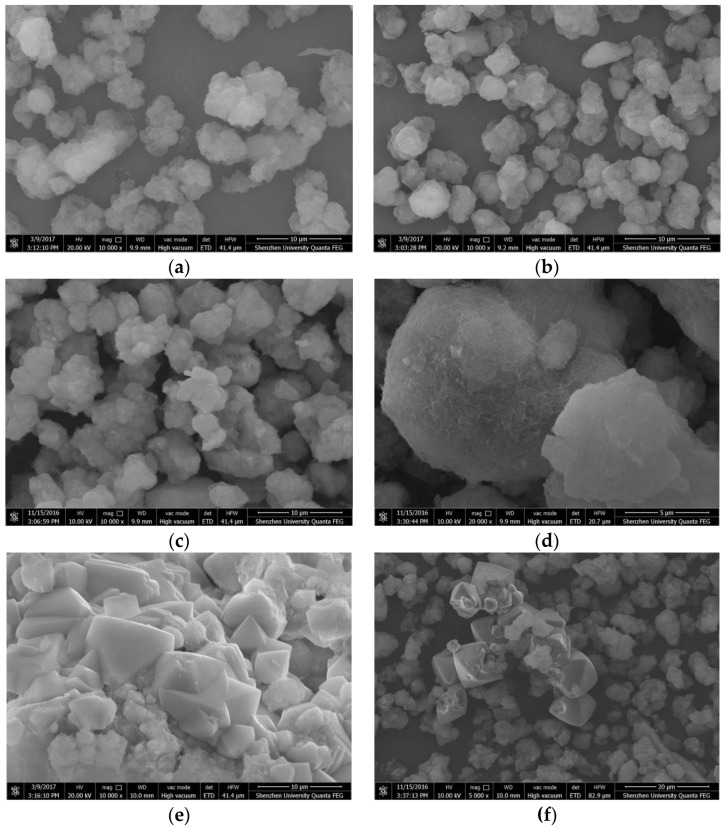
SEM images of the C-A-S-H system products. (**a**) CASH1.0_R_7d; (**b**) CASH1.0_60_7d; (**c**) CASH1.0_95_7d; (**d**) CASH1.0_95_7d; (**e**) CASH2.0_60_7d; (**f**) CASH2.0_95_7d.

As shown in Figure 9, the amorphous C-A-S-H gels exhibited agglomerated flocculent particles in the SEM images. The diameter of the agglomerated particles was mostly in the range of 3–8 μm and the particle size and morphology of the C-A-S-H gels changed slightly for the different curing conditions. The particle diameters of the C-A-S-H samples at room temperature and at 60 °C were for the most part smaller than 5 μm. The surface of the C-A-S-H gel particles was smoother when curing occurred at 95 °C compared to curing at a low temperature and the agglomerated particles were fuller and denser. It is assumed that an increase in the curing temperature renders the amorphous C-A-S-H gels more compact. Figure 9f shows the katoite in the C-A-S-H samples, which is consistent with the octahedral morphology of the katoite minerals synthesized by Passaglia et al. [38]. The SEM images of the sample CASH2.0_60_7d (Figure 9e) show the partially octahedral crystals but the octahedral morphology is not as regular as in Figure 9f. The other shapes of the crystalline material are likely poorly crystallized katoite, carbonized C_4_AcH_11_, or CaCO_3_.

**Figure 10:**
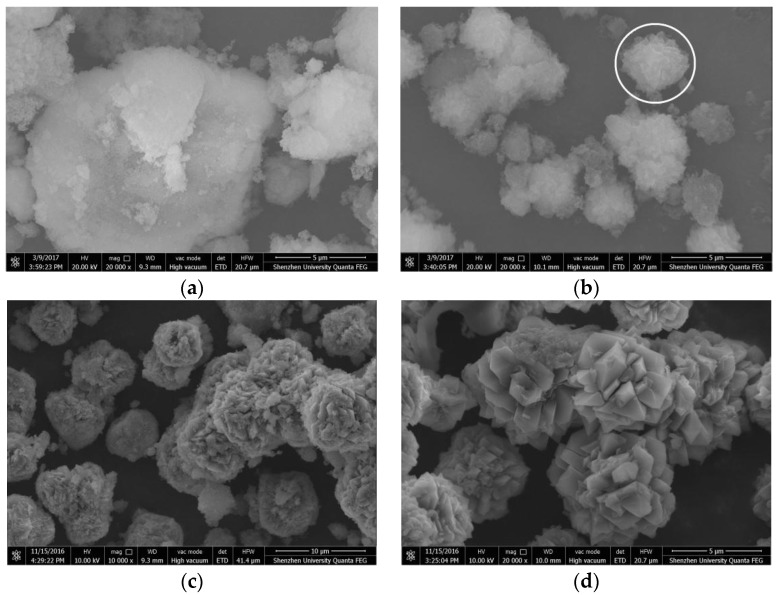
Scanning electron microscopy (SEM) images of the N-A-S-H system products. (**a**) NASH1.0_R_7d; (**b**) NASH1.0_60_7d; (**c**) NASH2.0_60_7d; (**d**) NASH1.0_95_7d.

Figure 10a–d depicts the crystallinity of the N-A-S-H system from low to high. For a lower Na_2_O/SiO_2_ ratio and at a low temperature, the reaction products are mainly amorphous N-A-S-H products (Figure 10a) and these products exhibit the irregular shapes and sizes of a flocculent morphology covered with a fine powder, which is unreacted silicon aluminum oxide. When the curing temperature was increased to 60 °C, most of the products remain irregularly flocculent and there was a small number of 2–3 µm diameter semi-crystalline particles, which were formed by massive stacking and exhibit fuzzy edges (as shown in Figure 10b in the circled area). The samples obtained by increasing the Na_2_O/SiO_2_ ratio to 2.0 at 60 °C are shown in Figure 10c; these semi-crystallized particles were clearer and the number of particles had also increased. Based on the XRD analysis results, these semi-crystallized particles were faujasite and zeolite-P with poor crystallinity. As shown in Figure 10d, the crystalline products cured at a high temperature were regular, interspersed with each other, and the diameter of the aggregates had increased to about 4–5 µm.

### 3.4. Transmission Electron Microscopy

**Figure 11:**
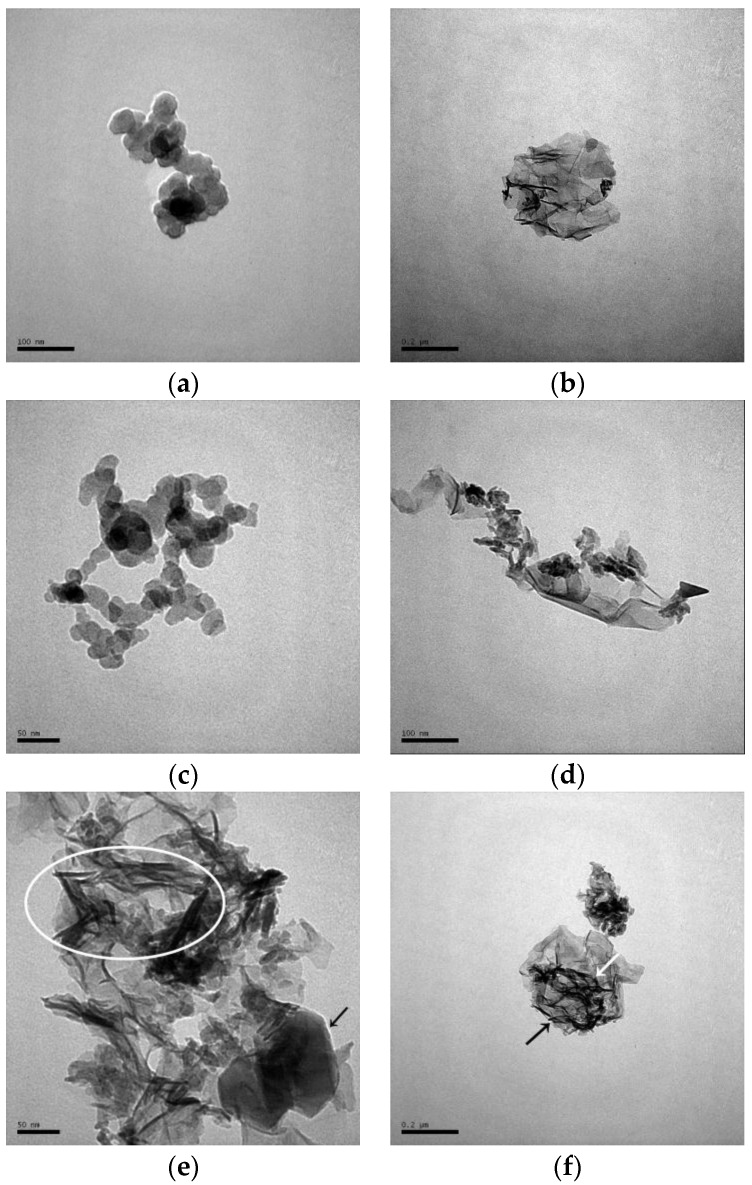
TEM images of the C-A-S-H system products. (**a**,**b**) CASH1.0_R_7d; (**c**,**d**) CASH1.0_60_7d; (**e**,**f**) CASH1.0_95_7d.

The sample CASH1.0_R_7d exhibited mainly two morphologies in the TEM; one was a chain of globular particles of about 40 nm with the globular particles overlapping each other; the other type of morphology was a flocculent structure, and the quantity of flocculent structure is more than the chain of globular particles in the sample CASH1.0_R_7d. As the curing temperature increased to 60 °C, the sample CASH1.0_60_7d also exhibited these two morphologies (Figure 11c,d) and the chain-like structure composed of the globular particles was more stretched than in the samples cured at room temperature. In addition, the overlap area of the globular particles was reduced and the globular particles were located end to end. The number of globular particles in the chain structure and the chain length increased. The sample CASH1.0_95_7d cured at a high temperature is shown in Figure 11e,f. This sample mainly exhibited the accumulation of the flocculent structure and, compared to the samples cured at a low temperature, the flocculent structure is much denser and there is no chain structure in the globular particles. Some fibrous structures with lengths of about 50 nm (as shown in Figure 11e,f in the circled and arrow marked areas) can be observed together with the flocculent structure. In addition, a small lamellar structure (as shown in Figure 11e in the arrow marked areas) can be observed at about 100 nm and this is the crystalline phase of the products.

**Figure 12:**
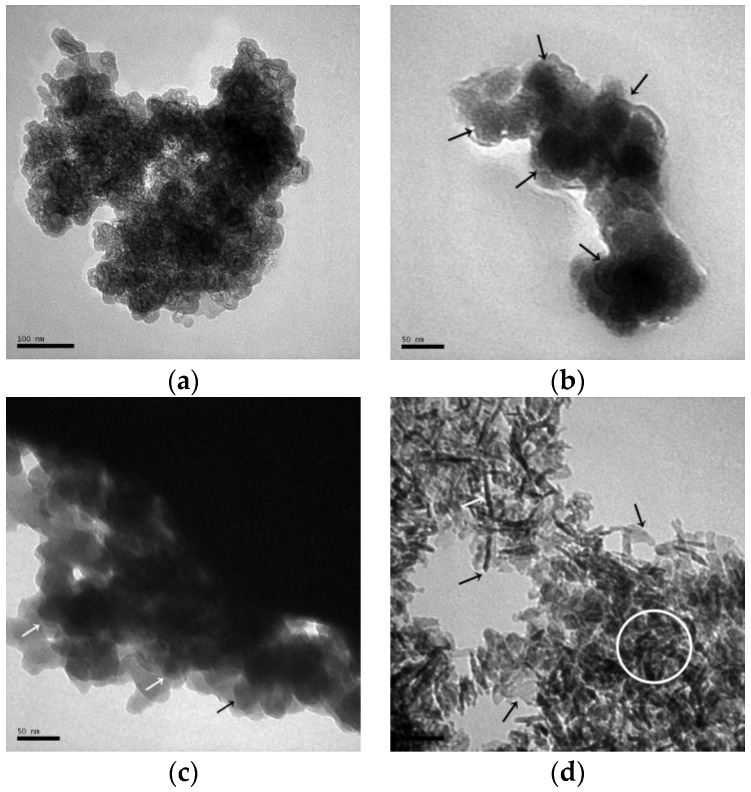
TEM images of the N-A-S-H system products. (**a**) NASH1.0_R_7d; (**b**) NASH1.0_60_7d; (**c**) NASH2.0_60_7d; (**d**) NASH1.0_95_7d.

Figure 12a is the TEM image of the sample NASH1.0_R_7d, which predominantly shows 30–40 nm globular particles gathered into a loose-mass structure with a porous surface. Combined with the XRD results, these are the amorphous N-A-S-H products. In sample NASH1.0_60_7d, the amorphous products were mainly composed of globular particles (as shown in Figure 12b in the arrow marked areas) that are similar to NASH1.0_R_7d (Figure 12a) but the structure was significantly denser and the pores on the surface of the globular particles almost disappeared. The lamellar crystalline N-A-S-H product (as shown in Figure 12c in the arrow marked areas) was observed at the edge of the sample in NASH 2.0_60_7d and overlapped with the globular particles. In the high-temperature cured sample NASH1.0_95_7d, it can be observed that the rod-like material at 100 nm overlapped with the irregular flakes (as shown in Figure 12d in the arrow marked areas) and a small amount of granular material (as shown in Figure 12d in the circled areas) was dispersed between the rod and the flakes. According to the results of the RIR quantitative analysis, the content of the crystallized products in this sample is greater than 70 wt.% and the materials with the rod and flaky morphology observed in the TEM image is zeolite-P. Based on the TEM images, the amorphous N-A-S-H products are predominantly globular and are similar to the TEM images of N-A-S-H gels synthesized using a sol-gel method as described by García-Lodeiro et al. [13].

### 3.5. Particle Size Distribution

**Figure 13:**
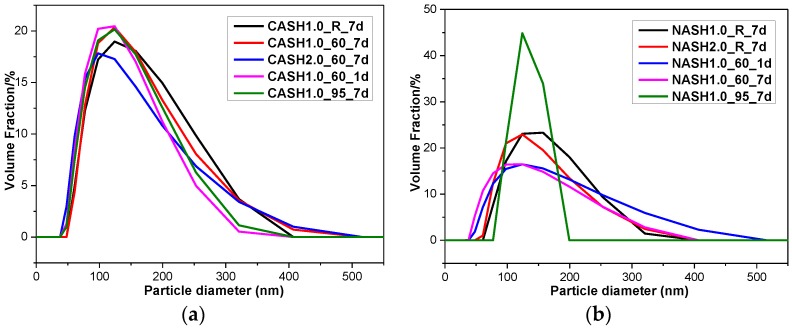
Particle size diameter distribution of the C-A-S-H and N-A-S-H products. (**a**) C-A-S-H system products; (**b**) N-A-S-H system products.

In order to further understand the particle size distribution of the C-A-S-H and N-A-S-H system products, some samples were selected for laser particle size distribution analysis. The results of the particle size distribution test and the average particle size analysis are shown in Figure 13 and Table 4, respectively.

The particle size distribution of the C-A-S-H system particles was relatively stable and the particle diameters were in the range of 100–300 nm (Figure 13), with an average particle diameter of 275 nm. The particle diameter distribution of the N-A-S-H samples was in the range of 100–300 nm with an average particle diameter of 285 nm. The results of the particle size distribution analysis are consistent with the results of the TEM observation. The products in the C-A-S-H system have a more stable phase composition according to the XRD and RIR methods results. The weight ratio of crystalline products in the C-A-S-H system is between 11.42 to 34.22 wt.%, but in the N-A-S-H system it is between 5.00 to 74.08 wt.%. This is the reason why the particle size distribution in the C-A-S-H system is more stable than that in the N-A-S-H system. According to the XRD and TEM analysis of sample NASH1.0_95_7d, more than 70 wt.% of this sample was a crystallized N-A-S-H product and the narrow range of the particle size distribution is related to the increase in the homogeneity of the product after crystallization.

## 4. Conclusions

C-A-S-H and N-A-S-H system particles with an average particle size smaller than 300 nm were synthesized by the hydrothermal method using nano-SiO_2_ and nano-Al_2_O_3_ as the silicon and aluminum sources, respectively. The hydrothermal synthesis method can directly reflect the influence of the chemical composition of the raw materials on the products without introducing other impurity ions, and the C-A-S-H and N-A-S-H systems can be regarded as simplified models of alkali-activated materials. In addition, the curing temperature and curing time have significant impacts on the composition and the microstructure of the products.

For the C-A-S-H system:

(1) The C-A-S-H gels were preferred products in the low-calcium C-A-S-H reaction system. With an increase in the CaO/SiO_2_ ratio and the curing temperature, katoite and other crystallized C-A-S-H products, as well as carbonated products such as C_4_AcH_11_, were observed and the types and contents of the crystallized products were related to the curing temperature and the content of excess Ca(OH)_2_.

(2) Increasing the temperature of the reaction system rendered the amorphous C-A-S-H gels’ structures more compact and the structure of the C-A-S-H gels was transformed from a chain structure of globular particles to a flocculent structure. There was no considerable change in the position of the bridging oxygen bond in TO_4_ when the curing conditions and the CaO/SiO_2_ ratio were varied.

For the N-A-S-H system:

(1) The N-A-S-H gels and other crystallization products, such as sodalite, faujasite, and zeolite-P were present in the N-A-S-H system. A high Na_2_O/SiO_2_ ratio and a high-temperature reaction system tended to convert the N-A-S-H products from amorphous to crystalline. According to the RIR quantitative results, the zeolite-P content in the high Na_2_O/SiO_2_ ratio and high-temperature curing system was more than 70 wt.%. The particle size distribution of the N-A-S-H system products are not as stable as that of the C-A-S-H system, which is caused by the great variations in the phase composition of the products.

(2) The N-A-S-H gels exhibited a structure consisting of globular particles and as the curing temperature was increased, the globular particles became denser and ultimately, short-rod and flaky crystalline products were formed. The increase in the Na_2_O/SiO_2_ ratio led to an increase in the number of non-bridging oxygen bonds in the N-A-S-H structure, which resulted in a shift of the principle band attributed to the asymmetric stretching of Si–O–T to a lower wavenumber as shown in the FTIR analysis. 

## Figures and Tables

**Table 1:** Mixing proportions used to prepared CaO-Al_2_O_3_-SiO_2_-H_2_O (C-A-S-H) and Na_2_O-Al_2_O_3_-SiO_2_-H_2_O (N-A-S-H).

Sample	CaO/SiO_2_	Na_2_O/SiO_2_	SiO_2_/Al_2_O_3_	Water/Solid Ratio
CASH1.0	1.0	-	2.0	8.0
CASH1.5	1.5	-	2.0	8.0
CASH2.0	2.0	-	2.0	8.0
NASH0.5	-	0.5	2.0	5.0
NASH1.0	-	1.0	2.0	5.0
NASH2.0	-	2.0	2.0	5.0

**Table 2:** Quantitative analysis results of C-A-S-H system using the reference intensity ratio (RIR) method.

Sample	Result (wt.%)	Sample	Result (wt.%)	Sample	Result (wt.%)
CASH1.0_R_1d	-	CASH1.0_60_1d	-	CASH1.0_95_1d	-
CASH1.0_R_3d	-	CASH1.0_60_3d	-	CASH1.0_95_3d	-
CASH1.0_R_7d	-	CASH1.0_60_7d	-	CASH1.0_95_7d	-
CASH1.5_R_1d	Ca(OH)_2_: 4.32	CASH1.5_60_1d	Ca(OH)_2_: 0.84	CASH1.5_95_1d	Katoite: 11.42
CASH1.5_R_3d	Ca(OH)_2_: 2.84	CASH1.5_60_3d	-	CASH1.5_95_3d	Katoite: 13.38
CASH1.5_R_7d	Ca(OH)_2_: 2.20	CASH1.5_60_7d	-	CASH1.5_95_7d	Katoite: 12.31
CASH2.0_R_1d	Ca(OH)_2_: 15.10	CASH2.0_60_1d	Ca(OH)_2_: 9.41	CASH2.0_95_1d	Katoite: 28.08
CASH2.0_R_3d	Ca(OH)_2_: 9.61	CASH2.0_60_3d	Ca(OH)_2_: 6.84	CASH2.0_95_3d	Katoite: 33.23
CASH2.0_R_7d	Ca(OH)_2_: 9.21	CASH2.0_60_7d	Katoite: 14.26	CASH2.0_95_7d	Katoite: 34.22

**Table 3:** Quantitative analysis results of N-A-S-H system using the RIR method.

Sample	Result (wt.%)	Sample	Result (wt.%)	Sample	Result (wt.%)
NASH0.5_R_1d	-	NASH0.5_60_1d	-	NASH0.5_95_1d	Zeolite-P: 36.40
NASH0.5_R_3d	-	NASH0.5_60_3d	-	NASH0.5_95_3d	Zeolite-P: 41.96
NASH0.5_R_7d	-	NASH0.5_60_7d	Faujasite: 11.62	NASH0.5_95_7d	Zeolite-P: 59.17
NASH1.0_R_1d	-	NASH1.0_60_1d	-	NASH1.0_95_1d	Zeolite-P: 56.91
NASH1.0_R_3d	-	NASH1.0_60_3d	Zeolite-P: 20.55	NASH1.0_95_3d	Zeolite-P: 73.82
NASH1.0_R_7d	-	NASH1.0_60_7d	Faujasite: 9.81/Zeolite-P: 29.19	NASH1.0_95_7d	Zeolite-P: 72.22
NASH2.0_R_1d	-	NASH2.0_60_1d	Zeolite-P: 5.00	NASH2.0_95_1d	Zeolite-P: 71.45
NASH2.0_R_3d	Sodalite: 5.11	NASH2.0_60_3d	Zeolite-P: 22.90	NASH2.0_95_3d	Zeolite-P: 71.68
NASH2.0_R_7d	Sodalite: 9.37	NASH2.0_60_7d	Zeolite-P: 34.19	NASH2.0_95_7d	Zeolite-P: 74.08

**Table 4:** Average particle diameter of selected samples.

Sample	Average Particle Diameter (nm)	Sample	Average Particle Diameter (nm)
CASH1.0_R_7d	295.7	NASH1.0_R_7d	304.5
CASH1.0_60_7d	291.5	NASH2.0_R_7d	285.7
CASH2.0_60_7d	270.2	NASH1.0_60_1d	308.3
CASH1.0_60_1d	253.1	NASH1.0_60_7d	261.3
CASH1.0_95_7d	264.7	NASH1.0_95_7d	259.5
